# Cranial Computer Tomography with Photon Counting and Energy-Integrated Detectors: Objective Comparison in the Same Patients

**DOI:** 10.3390/diagnostics14101019

**Published:** 2024-05-15

**Authors:** Anna Klempka, Alexander Schröder, Philipp Neumayer, Christoph Groden, Sven Clausen, Svetlana Hetjens

**Affiliations:** 1 Department of Neuroradiology, University Medical Centre Mannheim, Medical Faculty Mannheim, University of Heidelberg, 68167 Mannheim, Germany; 2 Department of Radiation Oncology, University Medical Centre Mannheim, Medical Faculty Mannheim, University of Heidelberg, 68167 Mannheim, Germany; 3 Department of Medical Statistics and Biomathematics, Medical Faculty Mannheim, University of Heidelberg, 68167 Mannheim, Germany

**Keywords:** photon counting, neurocranium, computer tomography, radiation dose

## Abstract

This study provides an objective comparison of cranial computed tomography (CT) imaging quality and radiation dose between photon counting detectors (PCCTs) and energy-integrated detectors (EIDs). We retrospectively analyzed 158 CT scans from 76 patients, employing both detector types on the same individuals to ensure a consistent comparison. Our analysis focused on the Computed Tomography Dose Index and the Dose-Length Product together with the contrast-to-noise ratio and the signal-to-noise ratio for brain gray and white matter. We utilized standardized imaging protocols and consistent patient positioning to minimize variables. PCCT showed a potential for higher image quality and lower radiation doses, as highlighted by this study, thus achieving diagnostic clarity with reduced radiation exposure, underlining its significance in patient care, particularly for patients requiring multiple scans. The results demonstrated that while both systems were effective, PCCT offered enhanced imaging and patient safety in neuroradiological evaluations.

## 1. Introduction

Computed tomography (CT) of the neurocranium is particularly challenging due to its clinical significance and the complex anatomy involved [1]. The imaging technique is expected to have enough penetrative power to traverse the notably thick cranial bones before it can accurately render the soft brain tissues. The human cranial bones undergo changes shaped by neurodevelopmental processes throughout an individual’s life [2]. Not only should soft brain tissue and bones be visualized, but underlying pathologies, such as solid brain tumors, or discrete changes in the parenchyma, such as microangiopathic lesions or even small bleeding spots, should be depicted [3,4]. Scientific research currently explores the possibilities of automating the detection of changes in the brain with the use of artificial intelligence (AI) to aid in the identification of abnormalities [5,6,7,8]. However, the authors of such studies strongly emphasize the need for human verification of AI conclusions.

In the last decades, cranial CT has shown improvements in overcoming the above-described challenges as well as in producing decent image quality. The image quality is a result of the device settings related to radiation [9,10]. Radiation may have an impact on certain diseases, and minimizing its exposure to the utmost necessary extent has been a priority in medicine, especially in pediatric care [11,12,13]. Furthermore, image quality is significantly affected by the detector’s performance, and the final image presented to the radiologist is shaped by the process of image reconstruction. This includes factors like kernel settings and slice thickness, which play crucial roles in determining the clarity and detail of the resulting images.

Recently, novel photon counting (PC) detectors have gained increased importance in clinical studies, and they have heralded a new era in CT. PCCT has demonstrated the potential to enhance image quality without compromising imaging performance in numerous radiological reviews as well as clinical studies [14]. These detectors are known for reducing radiation exposure while improving image quality [15,16]. Despite this advancement, many high-end, so-called energy-integrated detectors (EIDs) continue to be widely used in radiological check-ups due to their effective performance. The difference between PC technology and energy-integrated detectors is based on their signal processing. Specifically, photon counting technology allows for more precise energy resolution, reduced noise, and improved contrast detection. This contrasts with energy-integrated detectors designed to measure the total energy deposited over a period of time, which may potentially lead to less precise imaging, especially in low-contrast areas [17,18]. A notable limitation of energy-integrated detector systems is caused by their reliance on an intermediary step to gauge the energy of X-ray photons. This involves utilizing a scintillator, which converts the incoming X-ray photons into visible light prior to its conversion into electrical signals. Furthermore, the output signal amalgamates the total energy from all of the X-ray photons without providing precise details regarding their individual quantities or distinct energy values. PCCT facilitates the precise quantification of every photon directly at the detection stage.

The requirement to produce clinically useful images is beyond any doubt. Many reviews present an optimistic outlook on novel PC detectors, highlighting their potential [18]. Clinically, there are studies demonstrating the capability of these detectors to accurately image small parts of the middle ear or lamina cribrosa based on small cohorts, yielding promising results [19,20]. Every CT scan, despite the detector, is characterized by a radiation dose associated with mAs and kV, as well as specific aspects that produce an image, such as the pitch factor, the rotation time, and reconstruction patterns. The CT protocols at our institution are standardized, and they are routinely and regularly checked during the constancy testing of every device, regardless of the detector type. However, each device, due to the advancements in technology affecting CT images, differs from the rest. Thus, there are many objective criteria for imaging that can be considered when comparing CT scanners.

The capability to obtain detailed imaging and to differentiate between white and gray matter is crucial for a thorough assessment of the neurocranium. The assessment includes many disorders that are important for patient management [21]. In the evolving landscape of medical diagnostics, Magnetic Resonance Imaging (MRI) is an alternative that provides a non-ionizing radiation method for detailed anatomical and pathological assessments. This technology allows for repeatedly conducting examinations without the adverse effects associated with cumulative X-ray radiation exposure. However, the utility of MRI is not without limitations. Its effectiveness is moderated by challenges in depicting bone structures, differentiating ossified changes, and identifying minor hemorrhages [4]. These limitations underscore the necessity of a comprehensive evaluation of the patient’s suitability for MRI, taking into account the specific contraindications related to the magnetic field employed in this modality or the patient’s condition. Despite these challenges, the complementary roles of MRI and CT scans in the diagnostic process cannot be overstated, particularly in complex cases and in the refinement of differential diagnosis procedures. The synergistic use of MRI and CT scans enhances the diagnostician’s ability to distinguish between varied pathological states, thus leveraging the strengths of each imaging modality to provide a more accurate and holistic understanding of the patient’s condition.

The primary goal of the CT technique in neuroradiology is to generate images of sufficient quality to accurately identify pathology while administering the lowest possible dose of radiation to achieve the necessary diagnostic clarity. Our study aimed at an objective assessment of the advancements in detector technology and their impacts on patient care with a special emphasis on minimizing radiation exposure.

## 2. Materials and Methods

### 2.1. Patients

This retrospective study analyzed 158 scans from 76 patients (31 females, 45 males), aged between 19 and 91 years with a median age of 72 and an average age of 69. Each retrospective PCCT scan was compared with scans from the same patients that were obtained using energy-integrated detectors. The inclusion criteria were complete calvarium depiction and the presence of another scan for the same patient obtained using an EID CT scanner. The exclusion criteria were the absence of an EID CT scan or a post-craniotomy status. Each CT scan was clinically indicated due to medical necessities, such as follow-up examinations, dementia evaluation, etc.

### 2.2. Scanners and Radiation Dose

The 76 CT scans were conducted using PCCT (Naeotom Siemens, Siemens Healthineers, Forchheim, Germany) and 82-EID CT scans (specifically, the Sensation 64–30 scan, Definition AS-19 scan, and Definition Flash-33 scan models from Siemens, Forchheim, Germany). Each scanner’s performance was evaluated based on the Computed Tomography Dose Index (CTDI) as well as the Dose-Length Product (DLP). Given the clinical scenario, we took into consideration the entire diagnostic imaging process. Every CT scan was performed by experienced technical personnel to maintain the optimal position of the patients in the scanner.

### 2.3. Imaging Specification

**Figure 1:**
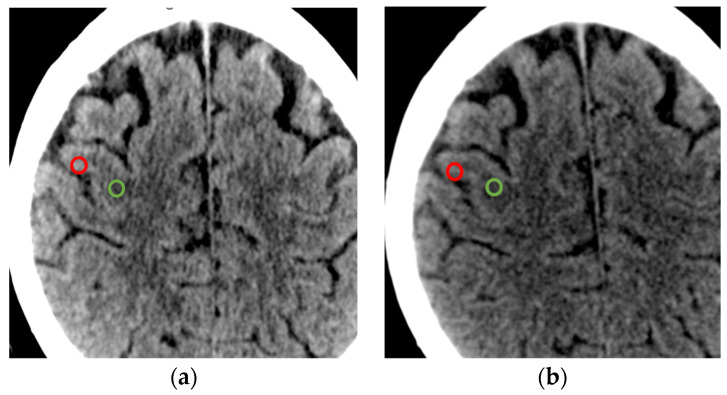
(**a**) PCCT scan; (**b**) EID scan with ROI markings of white matter in green and gray matter in red. Please note that macroscopically, there is no difference in the imaging from either scanner for the same patient due to the good quality of both scanners.

For each scanner, the clinically approved reconstruction protocol was used to image brain structures. The reconstructions, as mentioned in the introduction, varied in terms of noise and signal. To unify our assessment, we consistently employed clinically used protocols with clinically relevant increments. In this protocol, we assessed the mean Hounsfield Unit (HU) values and their standard deviation (SD) for gray and white matter using a Region of Interest (ROI) of approximately 15 mm^2^ and evaluated the contrast-to-noise ratio (CNR) and the signal-to-noise ratio (SNR), as conducted by an experienced radiologist (Figure 1). The homogenous reconstruction pattern with soft kernel, as well as comparable 4 mm slices, were used.

The CNR was calculated as the absolute difference between the mean signal intensities of gray matter and white matter, divided by the square root of the sum of the variances of the gray matter and the white matter (according to Michael et al. [22]). SNR is the signal intensity, measured in HU, divided by the standard deviation of mean pixel intensities within the white matter region or, respectively, the gray matter region. The Horos v3.3.6 open-source (https://horosproject.org; Horos Project, accessed on 1 March 2024) for the visualization and analysis of medical imaging data was utilized. For PCCT scanner imaging, the clinically approved polyenergetic reconstruction protocol was used. For other scanners, the clinically utilized reconstruction protocol in soft kernel was employed.

### 2.4. Statistics

All statistical calculations were performed using SAS software, release 9.4 (SAS Institute Inc., Cary, NC, USA). The methods were compared through paired *t*-tests or the Wilcoxon test. The prerequisite of paired tests is that the differences are normally distributed. The normal distribution of the differences was tested using the Shapiro–Wilk test. If this test was significant, the Wilcoxon test was calculated; otherwise, the paired *t*-test was used.

- For the comparison of the CTDI between the scanners PCCT and EID, the Wilcoxon test was used.
- For the comparison of the DLP between the scanners PCCT and EID, the Wilcoxon test was used.
- For the comparison of the SNR between the scanners PCCT and EID, the paired *t*-test was used.
- For the comparison of the CNR between the scanners PCCT and EID, the paired *t*-test was used.

For each parameter, quantitative approximately normally distributed parameters are presented by mean values, and those values are compared to each other as well as the standard deviation. Qualitative data are described by their absolute and relative frequencies and compared with each other as well. A *p*-value of less than 0.05 was considered statistically significant.

## 3. Results

### 3.1. Radiation Dose: Computed Tomography Dose Index (CTDI)

**Figure 2:**
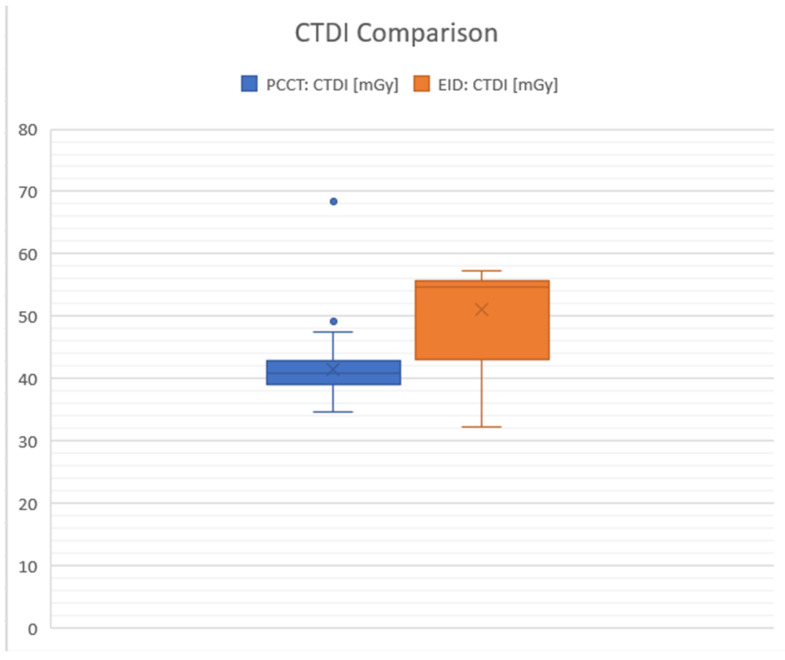
Graphical comparison between photon counting detectors (PCCTs) shown in blue and energy-integrated detectors (EIDs) in milligray (mGy). The graphic highlights the differences in the average Computed Tomography Dose Index (CTDI) values. The boxplot visually contrasts the CTDI values for PCCT and EID CT. It reveals that the median CTDI for EID is higher compared to PCCT, and EID’s interquartile range is narrower, suggesting that its CTDI values have less variability than those of PCCT. The maximum CTDI values are observed in PCCT, while the minimum values are found in EID. Whiskers: these lines extend from the box edges to the smallest and largest values in the dataset, excluding any outliers. For the blue box representing PCCT, the whiskers range from approximately 34.6 to 68.3 mGy. In contrast, the whiskers for the orange box representing EID extend from approximately 32.3 to 57.3 mGy. Boxes: these delineate the interquartile range (IQR), encompassing the middle 50% of the data. The blue box stretches from roughly 39.1 to 49.1 mGy. Meanwhile, the orange box spans from about 42.9 to 50.98 mGy. Horizontal line inside boxes: this line represents the median value within the dataset. For PCCT, the median is approximately 41.6 mGy, indicated by the line within the blue box. For EID, the median is around 50.98 mGy, also marked with an “X”.

For the PC detectors, the average CTDI was 41.56 milligray (mGy), with a standard deviation (SD) of 16.67, indicating some variation in the dose across different scans or settings. In contrast, the average CTDI for the energy-integrated detectors was higher at 50.99 mGy, with a lower variation (SD: 7.09). The difference in mean CTDI between the two types of detectors (PCCTDI and EID CTDI) was statistically significant at *p* < 0.0001 with a value of −9.43 mGy (95% confidence interval: −11.35 to −7.51), indicating that PC detectors, on average, used less of a radiation dose compared to EID detectors (in percentage, the difference between the mean values was 18.5%) (Figure 2).

### 3.2. Radiation Dose: Dose-Length Product (DLP)

**Figure 3:**
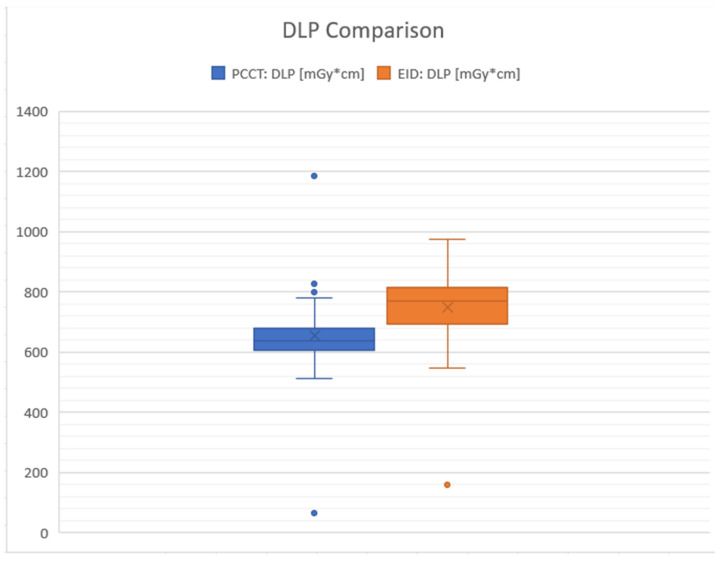
The boxplot contrasts the Dose-Length Product (DLP) in milligray–centimeters (mGy*cm) between photon counting CT (PCCT) in blue and energy-integrated detectors (EID) in orange. For PCCT, the interquartile range (IQR) spans from approximately 658.57 to 826 mGy*cm, with a median value indicated by the line inside the box at approximately 781 mGy*cm. Notably, there is an outlier at 1184 mGy*cm, which is significantly higher than the rest of the data points. The whiskers extend from about 512 to 1184 mGy*cm, defining the overall spread of the PCCT data. In comparison, EID’s IQR ranges from roughly 679.75 to 748.15 mGy*cm, with a median value marked by the “X” at approximately 769.87 mGy*cm. The EID data also include an outlier at 160 mGy*cm, which is well below the lower quartile. The whiskers for EID show data points ranging from approximately 547.51 to 974.27 mGy*cm. This comparison indicates a wider spread of values for PCCT, while EID presents a narrower distribution of DLP values with the median closer to the third quartile, suggesting a higher average DLP for EID than for PCCT.

In terms of the DLP, which considers the total radiation dose over the scanned volume, the mean for PC scans was 658.57 mGy*cm (with SD: 106.11), while for EID CT it was 748.15 mGy*cm (with SD: 111.24). The mean difference in DLP between PC and EID detectors was also statistically significant (*p* < 0.0001) at −89.58 mGy*cm (95% confidence interval: −120.88 to −58.27), further supporting the finding that PC detectors tend to result in lower radiation exposure than EID detectors. In summary, the results show that PC detectors are associated with significantly lower radiation doses (both CTDI and DLP) compared to EID in CT scans of the neurocranium (approximately 12% lower for DLP), suggesting potential benefits in reducing patient radiation exposure (Figure 3).

### 3.3. Imaging Specification: Comparison of the SNR (Signal-to-Noise Ratio)

In a detailed assessment of the signal-to-noise ratio (SNR) within both white and gray matter using CT, significant differences were observed between PCCT and EID CT technologies. For white matter, the average SNR recorded with PCCT was 14.15, with an SD of 3.22, compared to an average SNR of 10.35 (SD: 3.15) for EID CT. This yields a statistically significant mean difference of 3.81, with a 95% confidence interval (CI) ranging from 2.79 to 4.83, indicating the superior performance of PCCT in delineating white matter. Similarly, in the gray matter, PC CT demonstrated a higher mean SNR of 17.29 (SD: 4.53), while EID CT showed a mean SNR of 12.83 (SD: 5.16). The mean difference between the two technologies was 4.46 (approximately 26%), with a 95% CI of 3.08 to 5.85, further underscoring the enhanced capability of PC CT over EID CT in providing clearer, more distinct imaging of gray matter.

### 3.4. Imaging Specification Comparison of the CNR (Contrast-to-Noise Ratio)

**Figure 4:**
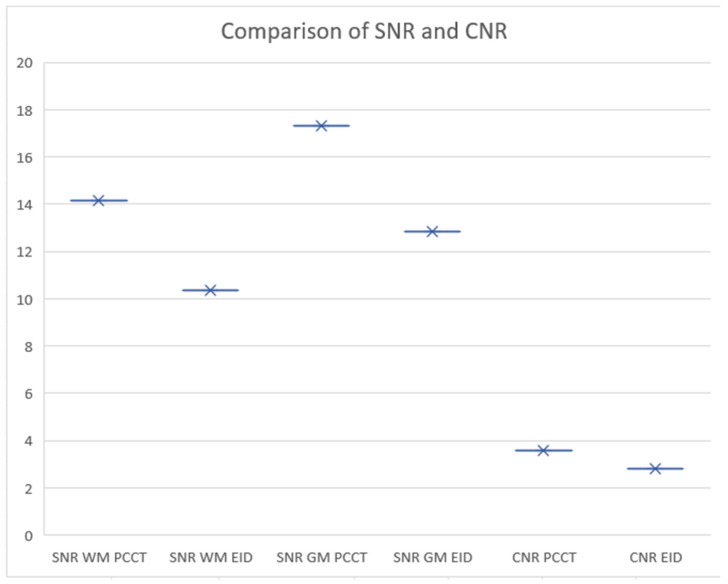
Comparison of signal-to-noise ratio (SNR) and contrast-to-noise ratio (CNR) in photon counting CT (PCCT) and energy-integrated detectors CT (EID CT). The graph illustrates SNR values for white matter (WM) and gray matter (GM) using both PCCT and EID technologies, as well as the CNR values achieved with each technique. The SNR for WM and GM is depicted by the X marks, with horizontal error bars representing the range of measurements. The comparison indicates that WM SNR is higher in PCCT compared to EID. For CNR, PCCT demonstrates a higher value than EID, suggesting better tissue contrast differentiation with PC technology. The data collectively suggest that PCCT may offer superior noise management and tissue contrast, potentially leading to more accurate diagnoses.

The analysis of the contrast-to-noise ratio (CNR) between PCCT and EID CT revealed a significant difference. The PCCT exhibited a CNR of 3.60 with a standard deviation (SD) of 1.32, while the EID technology showed a CNR of 2.79 (SD: 1.26). The statistical analysis demonstrated a significant (*p* < 0.0001) mean difference of 0.81, with a 95% confidence interval (CI) ranging from 0.42 to 1.20. This indicates that PCCT technology provides a superior contrast-to-noise performance compared to EID, suggesting its enhanced ability to distinguish between different tissue types and to detect abnormalities with greater clarity. A higher CNR (approximately 22.5% between mean values) indicates a better distinction between the gray matter and the white matter, which can be crucial for detecting abnormalities in medical images (Figure 4).

## 4. Discussion

The favorable perceptions of new technologies in clinical settings require a re-evaluation of their actual benefits beyond initial subjective assessments. The tendency for positive subjective analysis should be tempered with more objective studies that demonstrate the advantages of PCCT imaging. This study was designed to assess the clarity and diagnostic quality of CT scans with an emphasis on objective imaging characteristics, such as the differentiation between gray and white matter. A thorough understanding of normal anatomical states is imperative for accurate comparison against pathologies.

Several limitations must be acknowledged in our study. A notable consideration is that the ROI measurements were not automated but conducted manually by a professional with over a decade of expertise in neuroradiology. This introduces a subjective element to our quantitative analysis, which cannot be completely objectified. The accuracy and consistency of ROI placement significantly influence the reliability of HU values and, consequently, the computed CNR and SNR values.

Furthermore, the objective comparison of radiation doses administered during the CT scans is intrinsically affected by the attending technical personnel [23]. In our clinical setting, the staff meticulously ensure proper patient positioning on the CT scanner table for head examinations. While this reflects the clinical reality and the attentiveness of the staff to achieve optimal imaging outcomes, it could be misconstrued as a limitation. However, these practices are integral to the clinical workflow and are not unique to this study, representing a standard level of care in neuroradiologic imaging. The human element in both ROI assessment and patient setup is an inherent aspect of clinical practice, reflecting real-world circumstances rather than a flaw in the study’s design. Nonetheless, these factors underscore the importance of considering human-related variables when interpreting the results and their potential.

It is important to highlight the uniform application of radiation settings across adult patient demographics in PCCT in our study. We acknowledge the limitation that differentiation in X-ray exposure between adults and elderly patients was not fully explored. Addressing this issue comprehensively would likely require modifying standard practices, thereby ensuring more personalized and effective imaging assessments in PCCT. The concept of this study with a retrospective design included adult and elderly groups. This also reflects the aging German population, where an increasing number of older individuals receive medical attention.

A critical factor influencing the diagnostic process in medical imaging is the inherent subjectivity of radiologists’ judgments. This subjectivity arises from a combination of individual experience, training background, expectations, and even personal biases towards certain diagnostic outcomes. Such variability exists in interpretation, particularly in complex cases involving gray and white matter differentiation, bleeding, and stroke identification, highlighting the nuanced nature of radiological analysis. Subjectivity in judgment can both enrich and complicate the diagnostic process. On the one hand, experienced radiologists bring a wealth of knowledge and intuition to their interpretations, often drawing on subtle cues in images that less experienced practitioners might overlook. This depth of insight can be invaluable in diagnosing challenging cases where objective measures, such as SNR and CNR, may not fully capture the nuances of the condition. From the other side, our findings highlight the pivotal role of SNR and CNR as key indicators of imaging quality significantly influenced by the chosen reconstruction protocol and radiation levels [24]. Objective assessments revealed that imaging clarity, which is crucial for delineating gray and white matter as well potentially detecting bleeding and identifying strokes, varies significantly with the technical parameters of SNR and CNR. These factors are inherently dependent on the reconstruction method and the radiation dosage, affirming that optimizing these parameters can enhance diagnostic accuracy and patient safety [25]. The use of AI can also be more efficient in achieving higher image clarity with a lower signal-to-noise ratio and a higher contrast-to-noise ratio.

The method of evaluation in imaging studies often employs the Likert scale, a tool widely used across research to compare various imaging techniques [26,27]. However, this approach carries an inherent subjectivity, as radiologists’ judgments can differ based on their experience and familiarity with different reconstruction techniques. Objectivity can be enhanced through the integration of artificial intelligence, yet, at present, the most effective results are observed when the subjective perspectives of human evaluators are combined with machine learning algorithms [28]. This synergy between human insight and technological precision reinforces the evolving nature of diagnostic imaging, aiming for a balance that leverages the strengths of both human and artificial evaluators for optimal outcomes.

In the medical field, where CT scans are common diagnostic tools, the emphasis on radiation safety cannot be overstated. The necessity of radiation protection particularly escalates after a clinical examination, underscoring its critical role [29]. There is variability in the radiation dose delivered to patients, which is contingent on the specific scanner and the protocol used [30,31]. Strauss at al. and Smoll et al. have also emphasized that radiological protection is crucial across all body regions [32,33]. It is vital to acknowledge that imaging the brain, which is encased by the skull bones, requires precise radiation dosing to yield meaningful results. Unlike other body parts where ultrasound might replace CT scans for certain assessments, head imaging necessitates specific considerations due to the skull’s density. In Germany, adherence to standardized radiation doses for diagnostic CT scans is a regulated standard, and any deviation from these standards must be accompanied by a technical justification [23]. Decisions regarding the use of CT scans often involve weighing the benefits of detecting treatable pathologies or performing necessary follow-ups against the risks of radiation exposure. In many cases, the advantages of conducting a CT scan outweigh these risks.

This study’s findings regarding the superior image quality and reduced radiation doses of PCCT compared to EID in cranial CT imaging may have profound implications for patients with various neurological pathologies, such as tumors and ischemic conditions. For patients with tumors, the enhanced contrast-to-noise and signal-to-noise ratios provided by PCCT could lead to better differentiation between tumor tissue and surrounding healthy parenchyma. This precision is crucial for accurate diagnosis and treatment planning and for monitoring the effectiveness of therapies. For instance, in cases where surgical intervention is considered, detailed imaging helps in mapping out a safe surgical approach that minimizes the risk to vital brain structures. In conditions like ischemia, where early and precise detection of infarcted areas is essential, the high-quality imaging of PCCT can be particularly beneficial, potentially leading to timely medical interventions that can significantly improve patient outcomes.

The imaging clarity demonstrated in this study extends to other regions of the neurocranium not covered in this research, showing very positive results in comparison to other scanners. When discussing radiation dose, we ensure that visualization is not compromised, as exemplified by the study by Klempka et al., which showcased the imaging of the lamina cribrosa [20]. The difference in radiation doses in the study of the lamina cribrosa was depicted (due to the varying lengths of scans) by comparing the dose per single layer and showing lower results by approximately 50% per slice of PCCT. Our present study presents a clinical approach by comparing the total dose delivered during neurocranium scanning, taking into account the entire scan length. This comparison revealed a reduction of approximately 12% in the mean DLP. It is essential to note that the scanners we compared with PCCT were high-quality and already had optimized radiation dose. Therefore, comparing our group of patients with those from studies using scanners other than high-end EID scanners could demonstrate even more positive results, as our findings have shown.

Moreover, the lower radiation doses associated with PCCT are particularly advantageous for patients who require multiple scans, such as those with chronic conditions or undergoing treatment for progressive diseases. Reducing radiation exposure without compromising image quality is critical in minimizing the risk of radiation-induced complications over time. The subjective evaluation of these improvements has been explored in depth by, for example, Pourmorteza et al. in 2017 [34]. This technology represents a pivotal shift towards safer diagnostic practices, marrying efficiency with enhanced patient safety not only in neuroradiological imaging.

## 5. Conclusions

Photon counting CT technology demonstrates a significant advantage in neurocranium imaging even in comparison to EID scanners from the latest generations. In summary, the introduction of photon counting technology in CT imaging marks a significant advancement in reducing radiation exposure for whole cranial CT scans by approximately 12% for DLP and 18.5% for CTDI without compromising image quality. This is evidenced by an SNR increase of approximately 26% and a CNR increase of 22.5%. Our findings highlight the considerable benefits of PCCT in enhancing the SNR for both white and gray matter. This suggests that PCCT has the potential to improve diagnostic accuracy by providing clearer and more distinct imaging contrasts, all while maintaining patient safety.

## Data Availability

The data presented in this study are available upon reasonable request from the corresponding author.
